# Baboons' Response Speed Is Biased by Their Moods

**DOI:** 10.1371/journal.pone.0102562

**Published:** 2014-07-25

**Authors:** Yousri Marzouki, Julie Gullstrand, Annabelle Goujon, Joël Fagot

**Affiliations:** Aix-Marseille Université & Laboratoire de Psychologie Cognitive - Centre National de la Recherche Scientifique, Marseille, France; Université Pierre et Marie Curie, France

## Abstract

The *affect-as-information* hypothesis (e.g., Schwarz & Clore, 2003), predicts that the positive or negative valence of our mood differentially affects our processing of the details of the environment. However, this hypothesis has only been tested with mood induction procedures and fairly complex cognitive tasks in humans. Here, six baboons (*Papio papio*) living in a social group had free access to a computerized visual search task on which they were over-trained. Trials that immediately followed a spontaneously expressed emotional behavior were analyzed, ruling out possible biases due to induction procedures. RTs following negatively valenced behaviors are slower than those following neutral and positively valenced behaviors, respectively. Thus, moods affect the performance of nonhuman primates tested in highly automatized tasks, as it does in humans during tasks with much higher cognitive demands. These findings reveal a presumably universal and adaptive mechanism by which moods influence performance in various ecological contexts.

## Introduction

When we are in a good mood, we see the world in a more friendly way, and our perception is more positive. Likewise, when we are in a bad mood, we tend to evaluate things around us in a negative way. Mood is the target of many streams of research in various psychological areas. The current study focused on the congruency between the tone of the affective states and the subsequent responses, concepts and memories activated or primed by the moods. The latter idea is at the heart of the “affect-as-information” (AAI) framework [1], [2], [3], [4], [5].

According to the AAI model, negative moods motivate individuals to engage in deep processing of information called *systematic strategies*, whereas positive moods limit such motivation in favor of more superficial information processing called *heuristics*. Moreover, happy moods increase reliance on general knowledge structures, whereas negative moods decrease such reliance. Consequently, positive affective states do not require any particular cognitive resources, and people in good mood care less about detail processing. Conversely, negative affective state may foster an effortful detail-oriented processing more suitable for handling extreme situations [6].

Previous studies with humans have confirmed the predictions of the AAI model with a variety of tasks from semantic priming to problem solving [7]. For example, when participants were presented with sad or happy music prior to three types of tasks: categorization, judgment, or lexical decision, priming effects were significantly facilitated following a positively induced mood in the three types of tasks [8]. Moreover, in problem-solving situations such as relating some of the problem dimensions to previous knowledge, participants in a positive mood succeed better compared to those who are in a negative mood, due to a stronger focus on item rather than relational cues. However, one key-feature of the AAI model is the subject's awareness of his/her mood activation either by externally or internally emotion-related stimuli. This awareness seems important to trigger the bias toward either *systematic* or *heuristic* strategies providing the valence of the mood [9].

Studying animal affective states was revealed to be pivotal for many areas, especially the development of psychoactive drugs. One study showed that rats housed in unpredictable conditions (with multiple negative interventions) were slower than rats in predictable conditions to respond to the food tone stimulus [10]. Rats even demonstrated fewer responses to ambiguous non-reinforced tones close to the positive reinforcement tone. In the same line, the frequency of stereotypic behaviors suggestive of a poor well-being in capuchin monkeys correlates with the frequency of pessimistic like judgments in a cognitive task [11]. These results were taken as evidence that such cognitive biases can be used as an indicator of animal affective state [12]. In animals, these indicators were shown to have about the same relevance and behavioral significance as with conscious affective states and verbal reports in humans [10].

Our study aimed at testing, for the first time in a nonhuman primate species, three straightforward predictions of the AAI model. They were that the reaction times (RT) should be (1) faster when baboons are in a positive mood but (2) should be slower due to deeper cognitive processing when the same animals are in a negative mood; (3) the neutral affective condition should provide intermediate results. Ethological observations of the baboons' moods and measurements of their response speed in a computerized task were combined to test these three hypotheses.

Thus far, prior studies have been performed with human participants and have typically employed mood induction and fairly complex cognitive tasks. The previous study with rats [10] used, besides experimentally induced affective states, a highly stressful associative paradigm inducing signs of clinical depression. In this context, the current study had the advantage to demonstrate that moods can have subtle effects in much less extreme affective states than in the experiments with rats [10]. Importantly, moods were not induced by experimental means but simply inferred from the affective valence of behaviors occurring spontaneously in the baboons' social context. Our study had therefore the additional advantage that neither an induction nor a rewarding or a punishing procedure was used.

## Method

### 1. Animals and their living conditions

We studied six male baboons from the CNRS Primate Research Center (Rousset-sur-Arc, France). Three of these baboons (Felipe, Dan, Pipo, age range: 3–16 years) belong to a social group of 24 individuals comprising adult females with their offspring(s) as well as three male adults. The other three (Articho, Barnabe, Cloclo: age range: 5–7 years) were from a smaller group of four young adult males. These two groups were raised in outdoor enclosures (700 m^2^ and 16 m^2^, respectively) connected to indoor animal areas (20 m^2^ and 12 m^2^, respectively). The latter provided shelters during bad weather and were used for sleeping. The enclosures contained various vertical and horizontal climbing structures for behavioral enrichment as well as stones of different sizes under which the monkeys enjoy retrieving insects and worms. Moreover, each enclosure was connected to either one (for the smaller group) or two (for the larger group) experimental booths (4×8 m) in which the monkeys can interact with computers providing food rewards. Baboons could freely enter and leave each experimental booth that was adjacent to the enclosure and accessible via opened doors. Therefore, it was unnecessary to capture the subject or to separate it from its social group in order to conduct the research. This procedure was aimed at preventing any advert effects that capture and social isolation may entail. That procedure based on the voluntary participation of the subjects reduces stress levels, as inferred from the significant decrease in salivary cortisol levels as well as the frequencies of stereotypies [13]. Baboons were neither water nor food deprived during the research. Water was provided *ad-libitum* within the enclosure. Monkeys received their normal ratio of food (fruits, vegetables and monkey chows) every day at 5 pm. The baboons were all born within the primate center. This research received approval (# 15-06022013) from the “Comité d'Ethique CE-14 pour l'Expérimentation Animale”.

### 2. Ethological observation of baboons' moods

Four experimenters trained on ethological screening techniques (instantaneous sampling techniques, see 14) reported during eight days which behavior each individual exhibited at the observation time (see the procedure below). The emotional valence of the behaviors expressed by nonhuman primates is often difficult to infer because a single behavior can have different meanings depending on the social and experimental contexts in which it is exhibited. To alleviate this difficulty, we chose to follow a data-driven approach to infer the affective value of each behavior.

**Figure 1:**
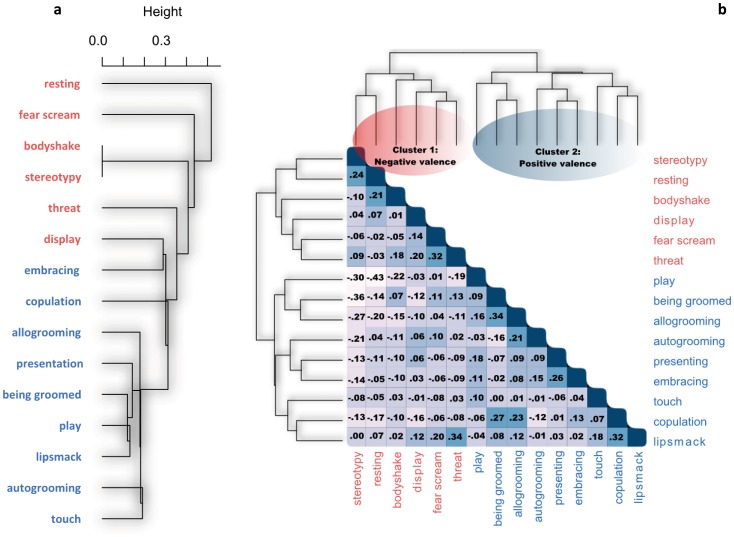
The dendrogram of the cluster solution based on the probabilities of co-occurrence (considering one minute time windows) of the valenced behaviors relative to each other, following their actual appearance on time over the eight days of observations (Fig. 1a). Rank correlation heat map with clusters' dendrogram based on the concordance matrix between the four raters of the 14 valenced behaviors. The reported values show the Kendall coefficients from negative (light blue) to positive (dark blue) rank correlation (Fig. 1b). These two analyses consistently revealed two main clusters: cluster 1 indicates the negative valence; cluster 2 indicates the positive valence.

Firstly we separated the most frequently observed behaviors (see the complete list in File S1) in two different groups by considering on one hand the affectively marked behaviors, regardless of their valence (positive or negative), and on the other hand behaviors with no affective feature. Secondly, in order to assign each affective behavior to a given valence, we extracted a matrix of the probabilities of co-occurrence (considering one minute time windows) of the valenced behaviors relative to each other, following their actual appearance on time over the eight days of observations. Our rationale for this analysis was that the behaviors with the same general affective valence, either positive or negative, might tend to emerge consistently over daily spontaneous activities of the subjects. A hierarchal clustering technique based on the centroid method was then applied on this co-occurrence matrix. Figure 1a shows the results of this cluster analysis. It suggests high likelihoods in grouping: *play, being groomed, allogrooming, autogrooming, presenting, embracing and touching a social partner, copulation and lipsmack* within the positively valenced cluster, and *stereotypy, body shake, display, fear scream and threat* within the negatively valenced cluster.

To further confirm the affective valence of these behaviors, we also created another matrix that contains the frequency of each valenced behavior observed by each rater, from which we extracted a concordance matrix calculated on the basis of Kendall coefficient as a symmetric measure of rank correlation between the four raters. Our rationale was to make sure that the above positively and negatively affective behaviors effectively belong to two distinct clusters, based on the four raters' coding. Likewise, this concordance matrix was submitted to a hierarchical cluster analysis. The results were straightforward and confirmed those reported in the first analysis (Figure 1b). Figure 1b showed two main clusters; the first reflects negatively valenced behaviors, whereas the second reflects positively valenced behaviors. Performance in the computerized task was therefore analyzed considering these two clusters of behaviors highlighted in Figure 1a and 1b, along with behaviors with no clear-cut emotional valence (*locomotor behavior, eating, drinking and object-directed exploratory behaviors*), which were considered as emotionally neutral.

### 3. The computerized RT task

The computerized task was administered via a 13 Automated Learning Devices for Monkeys (ALDM) [15], [16], which consisted in operant conditioning test devices equipped with 19 inches touch-screens and food dispensers. A critical feature of ALDM testing is that each baboon was automatically identified by a microchip controlling the initiation of the test program depending on the animal identity. Baboons could therefore participate to the task at will, without capture, and on a 24 hour basis. Grains of dry wheat were delivered as rewards. The computerized task is already described and detailed in a previous publication [17]. Briefly, each trial started with a fixation stimulus that the baboon had to touch to display the visual search display. The visual search display contained 8 white items (1 target and 7 distractors) on a black background. The task required the subject to search for a small T target (rotated 90°) embedded within configurations of L-shapes distractors of various orientations (randomly rotated 90°, 180° or 270°). Within trials, the configurations of the spatial location of the seven distractors were either predictive or non predictive of the target location. Twelve configurations of distractors were used for testing. Six of them were “predictive” and each was associated with a fixed target location. These “predictive” configurations could thus serve as cues for target detection. The six other “non predictive” configurations had no fixed relation between the location of the distractors and target location. Response times were defined as the time elapsed between the selection of the fixation stimulus and the touch response on one stimulus on the visual search screen display.

Training on the computerized task was proposed prior to the test. A first training phase required the discrimination of the rotated T-shaped target from a unique L-shaped distractor. This first phase continued until the baboon achieved 80% correct or more within two consecutive blocks of 100 trials. It required 383 trials on average (range: 300–500). In the second training phase, the target was embedded among three different “non predictive” configurations of seven distractors. Training sessions consisted of 72-trial blocks which were repeated until the baboon reached 80% correct or more in two consecutive blocks. An average of 396 trials was required in this phase (range: 288–576).

### 4. Experimental design and procedure

**Figure 2:**
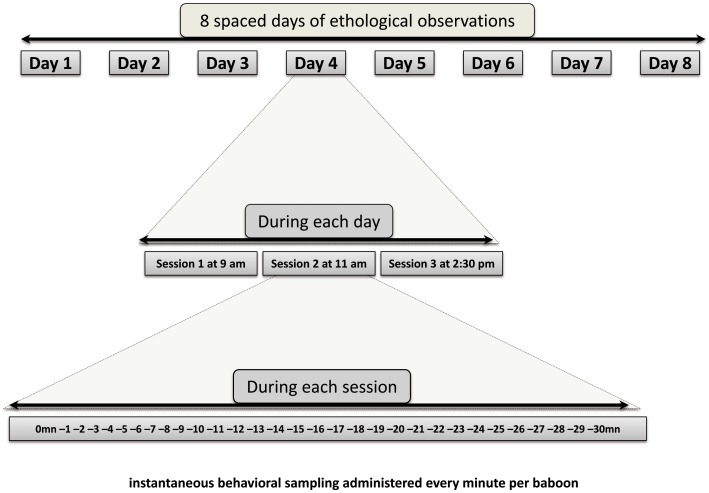
Timeline and frequencies of instantaneous behavioral sampling during the experiment.

Once the task became familiar after training, baboons were observed every minute (instantaneous sampling, see [14]) during three 30-min behavioral sessions per day during eight days (see Figure 2). This procedure has allowed the collection of a total of 4320 behavioral measures for the 6 baboons. As baboons had a permanent - unconstrained - access to the computers, performance in the computerized task and the social behaviors could be recorded simultaneously. A total of 2835 behaviors observed within the enclosure were followed by a voluntary participation to the computerized task within a time window of three minutes after the considered behavior. One advantage of the ALDM testing is that the baboons can produce extremely large numbers of computerized trials per day [18]. During the observation period, we could collect a total of 77533 computerized trials for the group (range: 4916–18834 per individual), and 7376 of them (range 796–1825) were performed within the three minutes time frame retained after each behavioral observation. With such database, we could examine how performance in the computerized trials varied depending on the category of (positively or negatively valenced) behaviors just presented within the enclosure.

The experimental design involves two hierarchical fixed factors: the daily *Session of observation* (9am, 11am and 2pm) nested within *Days of observation* (8 days) which may reflect training effects and also circadian fluctuation of cortisol [13]. They were crossed with two within-subjects factors: the *contextual cueing* (predictive vs. non predictive) and the *valence of the baboons' affective state* (positive, negative or neutral), see Figure 2.

Table 1 provides the distribution of RT trials as a function of *valence* and *contextual cueing*. As can be noticed during the test, non predictive trials (50%) were intermixed equivalently with predictive trials (50%) for each level of valence.

**Table 1:** The distribution of RT trials (number of trials and their corresponding percent) as a function of valence (positive vs. negative vs. neutral) and contextual cueing (non predictive vs. predictive).

	positive	negative	neutral
non predictive	488 (7%)	1040 (14%)	2183 (30%)
predictive	497 (7%)	997 (14%)	2171 (30%)

Since the emotional valence was spontaneously observed, observational data were highly unbalanced when crossed with the contextual cueing. We thus used Linear Mixed Effects (LME) technique for statistical analysis, with the six baboons being treated as a random factor. The reciprocal transformation was used for analyzing RT data [19].

## Results

**Figure 3:**
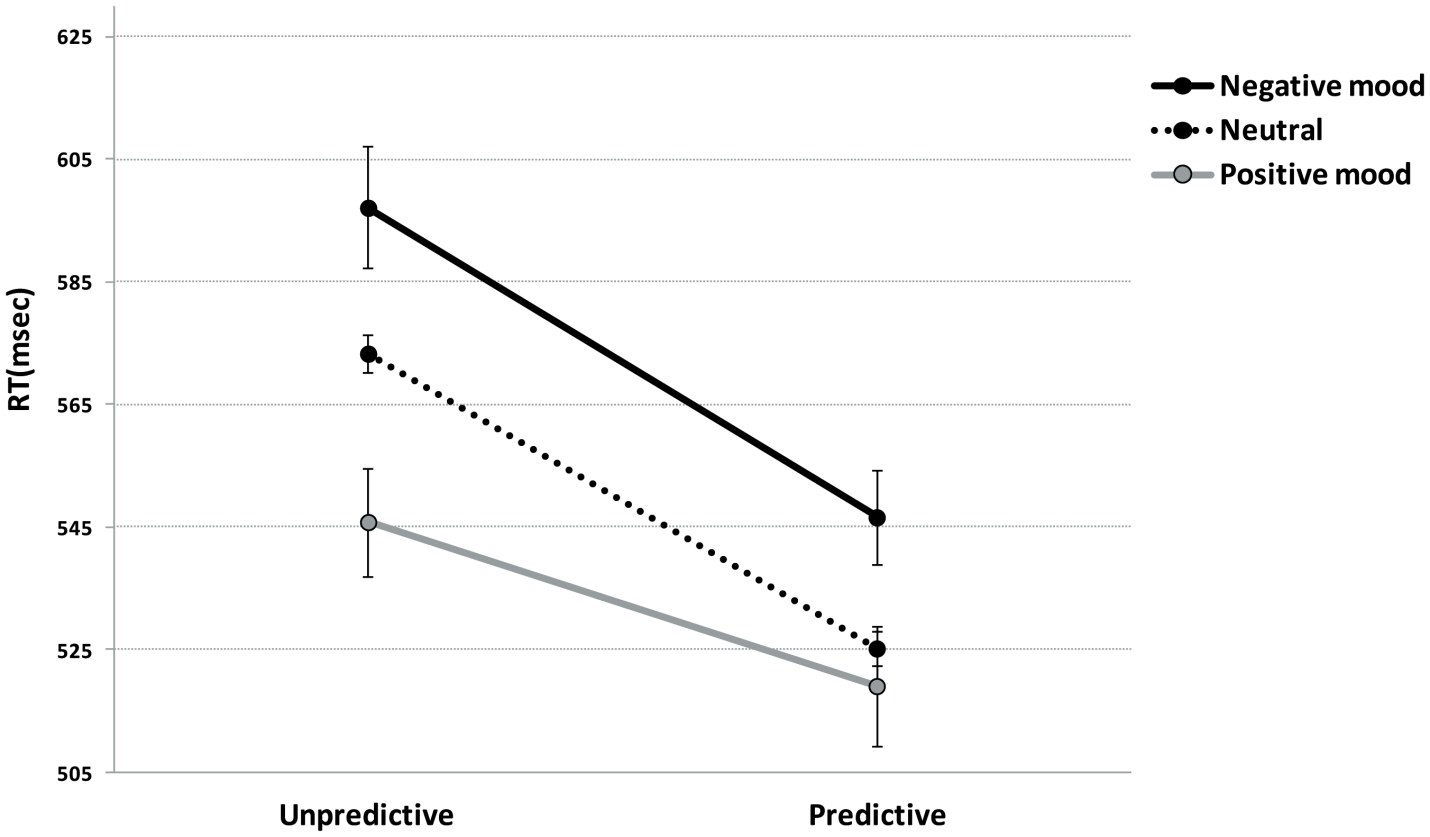
Mean RTs for trials following positive, negative, and neutral behaviors. Data are plotted as a function of affective state valence and contextual cueing. Error bars show standard errors.

Baboons reached an accuracy level of 96.0% correct on average (range: 94.2 to 98.8%) during the test. This task, which was already highly familiar prior to the ethological observation, was relatively easy to achieve. This extremely high performance precluded statistical analyses of the scores depending on the affective state and confirmed that the task became relatively easy after training. The negative affective state and the non predictive conditions were on the intercept of the following additive LME model: *Model 1* formula: *1/RT ∼ Days \ Session + Valence + Cueing | Subjects* (as random effect on the intercept). The “subjects” term fits random variation between animals in the experiment. We have tested in a separate analysis using the same LME technique the potential effect of social group (4 vs. 24 individuals) on baboon's RTs in this experiment. The results showed that there is no significant effect of the size of social group (p>.1) and no significant interaction with valence. The LME analysis showed a significant effect of affective state valence on RTs. Subjects were slower in the negative relative to the neutral, *t*(7374) = 2.7, *p* = .0062), and the positive moods condition, *t*(7374) = 3.2, *p* = .0015) where they showed the fastest RTs (see Figure 3). The difference between RTs in positive and neutral conditions was not significant, *t*(7374) = 1.4, *p* = .16. The results also replicated the previously shown main effect of contextual cueing [17]) with faster RTs in the predictive relative to the non predictive condition *t*(7374) = 10.6, *p*<.0001), irrespective of the valence of the baboons' affective state.

The interaction between contextual cueing and valence was tested in the following multiplicative LME model *Model 2* formula: *1/RT ∼ Days \ Session + Valence x Cueing | Subjects* (as random effect on the intercept) and was revealed to be not significant. The parsimonious explanation in our data favored a model without interaction between these two factors as shown by the khi-squared difference test between *Model 1* and *Model 2*, χ^2^(2) = 0.74, p = .6941.

## Discussion

The aim of the present study was to examine if baboons' affective state can influence their performance in a computerized task, following the predictions of the AAI model. We analyzed the trials that immediately followed spontaneously expressed emotional behaviors, which were characterized as being negative, positive or neutral. The results showed a significant effect of the affective state on baboons' response speed: RTs following negatively valenced behaviors are significantly slower than RTs of the neutral condition and those following positively valenced behaviors. The latter exhibited the fastest RTs.

The contribution of these findings is fourfold: firstly, they confirm, in an animal model, the predictions stemming from the AAI model [4], [5], a major human cognitive model of emotion; secondly, they validate a new methodology for studying the effect of emotional biases on animal cognition that relies on spontaneously (not induced) valenced behaviors; thirdly, it demonstrates that an highly familiar (presumably automatized) task produces the same results in an animal species as those of more challenging cognitive tasks in humans. Finally, it further demonstrates that the social context influence the behaviors during cognitive testing [20]. We suggest that this variation in cognitive processing after experiencing positive or negative emotions reflects a general adaptation mechanism present within the primate order (and potentially in other species, too) that increases the fitness of the behavior in a broad range of positive (e.g., social) or negative (e.g., predation) contexts. In that respect, the slowdown of the response times in negatively valenced contexts might prevent the production of impulsive and potentially poorly adapted responses in such contexts.

**Figure 4:**
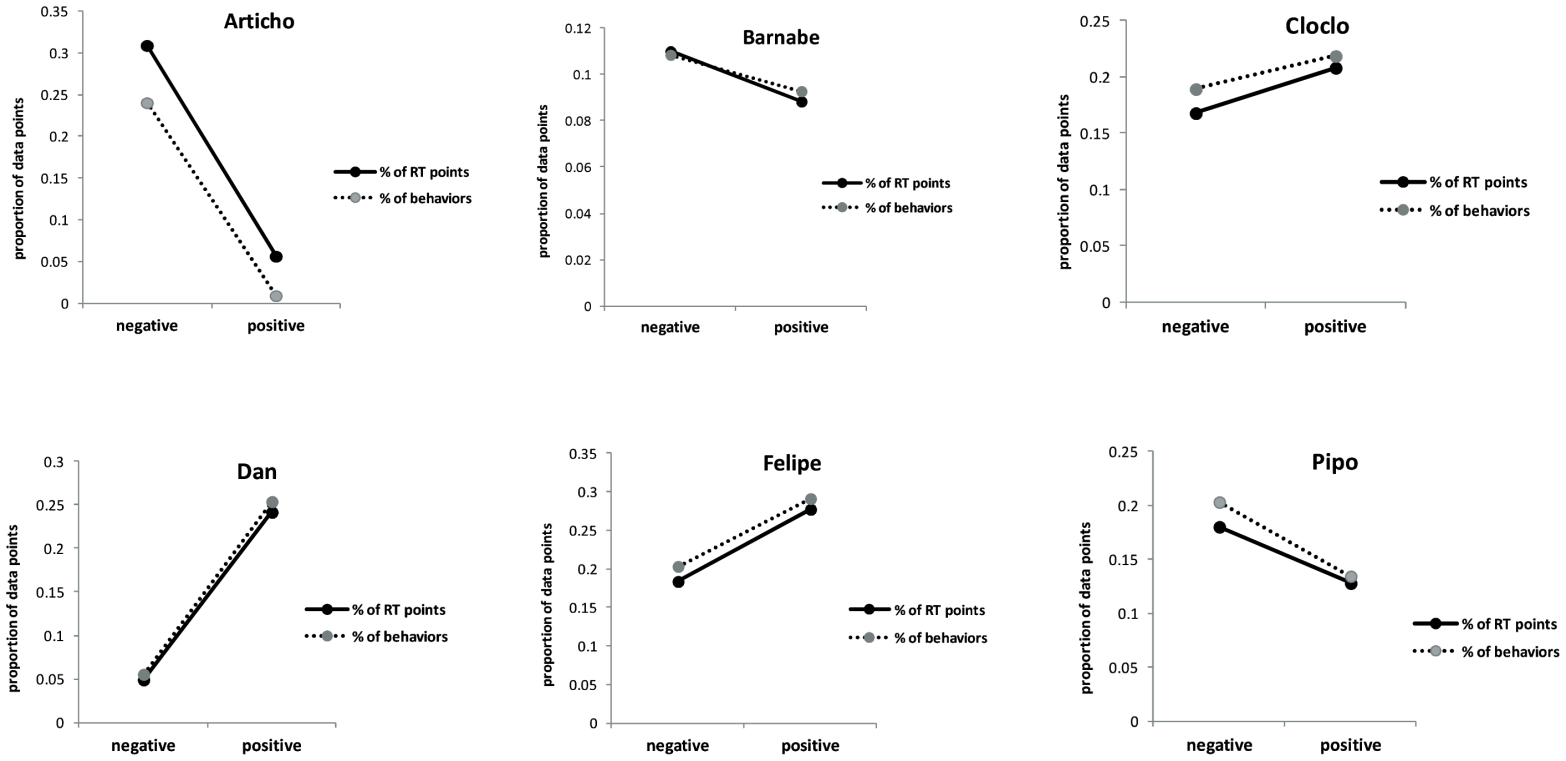
By-subjects trends of the proportion of data points as a function of positive and negative mood for both spontaneous valenced behaviors and the associated RTs. Note that the amount of computerized trials (% RT points) is about the same as the amount of expressed valenced behaviors (% of behaviors) either increasing or decreasing throughout.

Figure 4 illustrates the relation between the proportion of positive and negative behaviors observed within the enclosure, and the proportion of computerized test trials performed immediately after these positively or negatively valenced behaviors. Figure 4 reveals that the baboons had different emotional baselines, as some subjects (e.g., Dan, Felipe) mainly expressed positive social behaviors while others (e.g., Articho, Pipo) were more negatively biased. Figure 4 also shows a striking relation between the proportions of positively and negatively valenced behaviors and the corresponding proportion of test trials performed after these behaviors: The positively biased baboons interacted more frequently with the computers after positive social interactions and the negatively biased subjects performed more test trials after a negative social interaction. Interpretation of this effect remains unclear at this point. For instance, we can assume that interacting with the test computers helps the negatively biased subjects to calm down after a social conflict, while the positively biased subjects may use the computerized tests to further enhance welfare, but other hypotheses might be proposed too. Whatever the cognitive mechanism involved, this relation between emotional biases and the frequency of test trials promotes the idea that the animal behaves in a manner that is the most coherent with its own affective state.

One may have expected a speeding-up effect when subjects are in positive moods, but this was not found in the data. Earlier, we reported that ALDM testing [13] reduced salivary cortisol levels of baboons. Thus, our baboons already had slightly positive baselines, as they seem to enjoy computerized tasks which seemingly have an affective regulation on their behavior. According to the broaden-and-build theory of positive emotions [21], the latter widen the range of individuals' available thought-action repertoire. Positive stimuli tend to expand the attentional window and are typically associated with a greater and more durable distribution of resources, while these resources shrink with negative stimuli. The strong *cognitive depletion effect* of negatively valenced stimuli obtained in our study can be attributed to the larger effect that negative emotion has on our behavior both as individuals and also as a species [22].

In addition, these findings may also provide significant insight into forging grounds for new remedial strategies to avoid cognitive resources' depletion for clinical and subclinical populations. Clearly, these strategies should capitalize on the individual affective state during the remediation process. Finally, it is interesting to note that the contextual cueing had about the same importance as the affective state to influence the baboon's reaction times, and that the poorest performance was achieved when the baboons were in a negative mood. This latter result suggests that optimal performance in cognitive testing can only be achieved in nonhuman primates (and supposedly with other animals too) in test conditions favoring welfare. Our findings, therefore, call for a better understanding of animal welfare rooted in an ecological conception that includes affective states as part of animal's health in its natural housing conditions [23].

## Supporting Information

File S1 **Supplementary material.**
(DOCX)

Checklist S1 **The ARRIVE guidelines checklist.**
(DOC)
