# Supplementary material for: Baboons' Response Speed Is Biased by Their Moods
Source: PLoS One. 2014 Jul 25;9(7):e102562. doi: 10.1371/journal.pone.0102562 (PMC4111360; doi:10.1371/journal.pone.0102562)
Supplement: File S1 — Supplementary material. (DOCX) [file pone.0102562.s001.docx]

**Table S1. Probabilities of co-occurrences over time between behaviors. Note the asymmetrical feature of the matrix because of the time primacy constraint**

|  | **Valenced behaviors** | | | | | | | | | | | | | | |
| --- | --- | --- | --- | --- | --- | --- | --- | --- | --- | --- | --- | --- | --- | --- | --- |
| **n-1** | being groomed | coupling | bodyshake | Fear scream | display | embracing | allogrooming | play | lipsmack | threat | presenting | resting | autogrooming | stereotypy | touch |
| being groomed | 0.000 | 0.043 | 0.000 | 0.000 | 0.000 | 0.014 | 0.217 | 0.087 | 0.029 | 0.000 | 0.000 | 0.478 | 0.101 | 0.000 | 0.029 |
| coupling | 0.143 | 0.000 | 0.000 | 0.000 | 0.000 | 0.000 | 0.143 | 0.000 | 0.143 | 0.000 | 0.000 | 0.286 | 0.143 | 0.000 | 0.143 |
| bodyshake | 0.000 | 0.000 | 0.000 | 0.000 | 0.000 | 0.000 | 0.000 | 0.000 | 0.000 | 0.000 | 0.000 | 1.000 | 0.000 | 0.000 | 0.000 |
| fear sceam | 0.000 | 0.000 | 0.000 | 0.000 | 0.000 | 0.000 | 0.000 | 0.000 | 0.000 | 0.500 | 0.000 | 0.500 | 0.000 | 0.000 | 0.000 |
| display | 0.000 | 0.000 | 0.000 | 0.000 | 0.000 | 0.000 | 0.429 | 0.143 | 0.000 | 0.000 | 0.000 | 0.286 | 0.143 | 0.000 | 0.000 |
| embracing | 0.000 | 0.143 | 0.000 | 0.000 | 0.000 | 0.000 | 0.286 | 0.143 | 0.000 | 0.000 | 0.143 | 0.286 | 0.000 | 0.000 | 0.000 |
| allogrooming | 0.238 | 0.000 | 0.000 | 0.000 | 0.000 | 0.000 | 0.000 | 0.119 | 0.024 | 0.000 | 0.000 | 0.500 | 0.071 | 0.000 | 0.048 |
| play | 0.100 | 0.029 | 0.000 | 0.014 | 0.000 | 0.000 | 0.043 | 0.000 | 0.000 | 0.000 | 0.000 | 0.729 | 0.071 | 0.000 | 0.014 |
| lipsmack | 0.111 | 0.037 | 0.000 | 0.000 | 0.000 | 0.000 | 0.074 | 0.074 | 0.000 | 0.000 | 0.000 | 0.630 | 0.074 | 0.000 | 0.000 |
| threat | 0.500 | 0.000 | 0.000 | 0.000 | 0.000 | 0.000 | 0.000 | 0.000 | 0.000 | 0.000 | 0.000 | 0.500 | 0.000 | 0.000 | 0.000 |
| presentation | 0.167 | 0.000 | 0.000 | 0.000 | 0.000 | 0.000 | 0.000 | 0.000 | 0.000 | 0.000 | 0.000 | 0.667 | 0.167 | 0.000 | 0.000 |
| resting | 0.194 | 0.004 | 0.009 | 0.004 | 0.013 | 0.026 | 0.084 | 0.194 | 0.079 | 0.004 | 0.022 | 0.000 | 0.308 | 0.004 | 0.053 |
| autogrooming | 0.060 | 0.000 | 0.000 | 0.000 | 0.012 | 0.000 | 0.048 | 0.107 | 0.012 | 0.000 | 0.000 | 0.738 | 0.000 | 0.000 | 0.024 |
| Stereotypy | 0.000 | 0.000 | 0.000 | 0.000 | 0.000 | 0.000 | 0.000 | 0.000 | 0.000 | 0.000 | 0.000 | 1.000 | 0.000 | 0.000 | 0.000 |
| touch | 0.091 | 0.000 | 0.000 | 0.000 | 0.045 | 0.000 | 0.091 | 0.091 | 0.091 | 0.000 | 0.000 | 0.500 | 0.091 | 0.000 | 0.000 |

**Table S2. Descriptive statistics of the valenced behaviors exhibited by the baboons during the experiment along with the number of computerized trials following each behavior.**

| **Behavior** | **Description** | **Sum** | **Mean** | **Median** | **Range** | **Number of ALDM trials linked**  **to this behavior** |
| --- | --- | --- | --- | --- | --- | --- |
| allo grooming | the subject grooms another individual | 118 | 19.7 | 20 | 6-36 | 36 |
| auto grooming | the subject grooms itself | 123 | 20.5 | 18 | 6-46 | 117 |
| being grooming | the subject is groomed by another individual | 277 | 46.2 | 27.5 | 13-145 | 163 |
| bodyshake | shaking itself with ruffled fur | 2 | 1.0 | 1 | 1-1 | no RTs were matched |
| copulation | engaging in sexual intercourse. | 8 | 2.7 | 2 | 1-5 | 30 |
| display | hitting, crashing or shaking different objects or itself making noise | 9 | 1.8 | 2 | 1-3 | 41 |
| embracing | two monkeys are in the arms of each other, usually grunting | 12 | 2.0 | 1.5 | 1-5 | no RTs were matched |
| fear scream | shrill scream usually made during an attack by the victim | 1 | 1.0 | 1 | 1-1 | no RTs were matched |
| lipsmack | lips and tong in rythmic movement, directed to an indivual (usually use in presentation, or asking for something) | 28 | 4.7 | 4 | 2-8 | 129 |
| play | (social play=) fight simulation without vocalization (scream…) or just chattering. | 172 | 28.7 | 11 | 2-104 | 375 |
| presenting | an individual is approaching another and touch it lipsmaking | 6 | 2.0 | 2 | 1-3 | 135 |
| resting | inactive or sleeping | 973 | 162.2 | 176.5 | 75-270 | 1996 |
| stereotypy | repeatedly activity without aim, like pacing, rhythmic jumping, and rocking. | 1 | 1.0 | 1 | 1-1 | no RTs were matched |
| threat | Gaze eyebrow raising or slapping directed to an individual which is the target of the threat | 2 | 2.0 | 2 | 2-2 | no RTs were matched |
| touch | touching another individual | 26 | 5.2 | 3 | 1-11 | no RTs were matched |
| *neutral behaviors* | *locomotion, object-directed or other behaviors* | *1121* | *186.8* | *179* | *116-221* | 4354 |
| *invisible* | *not able to see the subject* | *1441* | *240.2* | *243* | *162-315* | - |
